# Star Allele-Based Haplotyping versus Gene-Wise Variant Burden Scoring for Predicting 6-Mercaptopurine Intolerance in Pediatric Acute Lymphoblastic Leukemia Patients

**DOI:** 10.3389/fphar.2019.00654

**Published:** 2019-06-11

**Authors:** Yoomi Park, Hyery Kim, Jung Yoon Choi, Sunmin Yun, Byung-Joo Min, Myung-Eui Seo, Ho Joon Im, Hyoung Jin Kang, Ju Han Kim

**Affiliations:** ^1^Seoul National University Biomedical Informatics (SNUBI), Division of Biomedical Informatics, Seoul National University College of Medicine, Seoul, South Korea; ^2^Department of Pediatrics, Asan Medical Center, University of Ulsan College of Medicine, Seoul, South Korea; ^3^Department of Pediatrics, Seoul National University College of Medicine, Seoul, South Korea; ^4^Seoul National University Cancer Research Institute, Seoul, South Korea; ^5^Center for Precision Medicine, Seoul National University Hospital, Seoul, South Korea

**Keywords:** 6-mercaptopurine, drug toxicity, variant burden, pharmacogenetics, pharmacogenomics, next-generation sequencing, Nudix Hydrolase 15 (NUDT15), Thiopurine S-Methyltransferase (TPMT)

## Abstract

*Nudix Hydrolase 15 (NUDT15)* and *Thiopurine S-Methyltransferase (TPMT)* are strong genetic determinants of thiopurine toxicity in pediatric acute lymphoblastic leukemia (ALL) patients. Since patients with *NUDT15* or *TPMT* deficiency suffer severe adverse drug reactions, star (*) allele-based haplotypes have been used to predict an optimal 6-mercaptopurine (6-MP) dosing. However, star allele haplotyping suffers from insufficient, inconsistent, and even conflicting designations with uncertain and/or unknown functional alleles. Gene-wise variant burden (GVB) scoring enables us to utilize next-generation sequencing (NGS) data to predict 6-MP intolerance in children with ALL. Whole exome sequencing was performed for 244 pediatric ALL patients under 6-MP treatments. We assigned star alleles with PharmGKB haplotype set translational table. GVB for *NUDT15* and *TPMT* was computed by aggregating *in silico* deleteriousness scores of multiple coding variants for each gene. Poor last-cycle dose intensity percent (DIP < 25%) was considered as 6-MP intolerance, resulting therapeutic failure of ALL. DIPs showed significant differences ( *p* < 0.05) among *NUDT15* poor (PM, *n* = 1), intermediate (IM, *n* = 48), and normal (NM, *n* = 195) metabolizers. *TPMT* exhibited no PM and only seven IMs. GVB showed significant differences among the different haplotype groups of both *NUDT15* and *TPMT* ( *p* < 0.05). Kruskal–Wallis test for DIP values showed statistical significances for the seven different GVB score bins of *NUDT15*. GVB*^NUDT15^* outperformed the star allele-based haplotypes in predicting patients with reduced last-cycle DIPs at all DIP threshold levels (i.e., 5%, 10%, 15%, and 25%). In *NUDT15*-and-*TPMT* combined interaction analyses, GVB*^NUDT15^* ^,^ *^TPMT^* outperformed star alleles [area under the receiver operating curve (AUROC) = 0.677 vs. 0.645] in specificity (0.813 vs. 0.796), sensitivity (0.526 vs. 0.474), and positive (0.192 vs. 0.164) and negative (0.953 vs. 0.947) predictive values. Overall, GVB correctly classified five more patients (i.e., one into *below* and four into *above 25% DIP* groups) than did star allele haplotypes. GVB analysis demonstrated that 6-MP intolerance in pediatric ALL can be reliably predicted by aggregating NGS-based common, rare, and novel variants together without hampering the predictive power of the conventional haplotype analysis.

## Introduction

6-Mercaptopurine (6-MP) is a commonly used drug in the maintenance therapy of pediatric acute lymphoblastic leukemia (ALL). Since patients have a potential to experience medication-induced life-threatening side effects including bone marrow suppression and hepatotoxicity, providing a tailored drug dosing regimen is essential in clinical practice (Vogenberg et al., 2010).

One of the strongest ways to determine initial 6-MP dose is an experimental assessment of potential for drug adverse reactions, such as severe neutropenia by monitoring 6-MP metabolite concentration or using *in vitro* activity profiles (Dubinsky et al., 2000; Ansari et al., 2002;; Cuffari, 2005; Bradford, 2011; Supandi et al., 2018). However, applying such methods into routine clinical practice for predicting drug-induced toxicity is still challenging because it is extremely time-consuming, expensive, and inefficient (González-Lama and Gisbert, 2016).

As recent studies have demonstrated the strong association between genetic polymorphisms and inter-individual variability in 6-MP dose intensity, approaches to predict drug tolerance on the basis of individual genomic profiles have arisen. The primary genetic determinant of thiopurine toxicity is *TPMT*, which plays a crucial role in identifying patients in need of treatment modification with reduced enzyme activity (Lennard, 2014). However, this has not been applicable to East Asian populations since the frequency of *TPMT* polymorphisms varies by ethnicity (Relling et al., 2013). Recently, a novel pharmacogenetic marker, *NUDT15*, has clarified its role in predicting thiopurine toxicity in Asian populations (Yang et al., 2014; Yang et al., 2015; Zgheib et al., 2016; Kakuta et al., 2017). Clinical Pharmacogenetics Implementation Consortium (CPIC) published an updated guideline for thiopurine dosing based on both *TPMT* and *NUDT15* genotypes using the star allele-based dose prediction method (Relling and Klein, 2009; Relling et al., 2018). This prevailing method provides therapeutic recommendations for dosing based on star allele genotypes. However, the utilization of star alleles in clinical practice has many obstacles that occur mainly due to 1) the extremely complex nomenclature system, 2) the limited resolution of phenotype prediction due to many unknown and uncertain function alleles, 3) ignorance of functional impacts of rare and/or novel variants, and 4) limited use in previously studied populations only (Robarge et al., 2007). Next-generation sequencing (NGS) challenges the conventional star alleles on the basis of genotyping technologies and clinical studies in case–control settings.

In the era of NGS, the comprehensive genotyping capabilities of NGS platform have enabled us to capture the true diversity of gene variation, and researchers propose alternative ways to predict individual intolerance towards a drug. One promising method is a gene-wise variant burden (GVB) scoring approach that can calculate gene-wise cumulative variant deleteriousness scores including common, rare, and even novel genetic variants for each gene (Lee et al., 2016). Here, we assessed the utility of GVB scoring method in quantifying the potential contributing effect of variants on enzymatic activity. By combining the clinically proven and well-established associations between the two genes, i.e., *NUDT15* and *TPMT*, and 6-MP dose intensity percent (DIP, actual/planned dose) as a clinical endpoint, we performed a comparison study of the conventional star allele-based haplotyping and GVB scoring methods for predicting the last-cycle 6-MP DIP as an indicator for 6-MP intolerance of ALL patients with *NUDT15* and/or *TPMT* deficiency. Overall, both star alleles and GVB showed significant correlations with 6-MP DIP values. Star allele-based haplotype groups showed significant correlation with GVB score groups. For predicting reduced last-cycle DIP values, GVB analysis outperformed the conventional star allele method for *NUDT15* and showed comparable result for *TPMT*. In *NUDT15*-and-*TPMT* combined interaction analyses, GVB*^NUDT15^* ^,^ *^TPMT^* outperformed star allele-based predictions [area under the receiver operating curve (AUROC) = 0.677 vs. 0.645] in specificity (0.813 vs. 0.796), sensitivity (0.526 vs. 0.474), and positive (PPV; 0.192 vs. 0.164) and negative (NPV; 0.953 vs. 0.947) predictive values. It is demonstrated that gene-wise evaluation of *in silico* deleterious variant score burden can be a useful method for predicting 6-MP intolerance in pediatric ALL patients, considering NGS-based common, rare, and novel variants concurrently while not hampering the predictive power of the conventional haplotype analysis.

## Materials and Methods

### Patients and Clinical Data Collection

A total of 298 Korean pediatric ALL patients with 6-MP treatment during maintenance therapy were recruited in the present study from two major teaching hospitals, i.e., Asan Medical Center (AMC) and Seoul National University Hospital (SNUH). Of the 298 subjects, 244 individuals who did not meet the exclusion criteria (i.e., relapse of the disease, stem cell transplantation, Burkitt’s lymphoma, mixed phenotype acute leukemia, infant ALL, or very high risk) were selected. All participants provided written informed consent. The study was approved by the AMC Review Boards and the SNUH Review Boards. The 6-MP dose per meter body surface area over a 12-week cycle was recorded. The maximum tolerated dose of 6-MP was defined as the dose at the last maintenance cycle for each patient. Patients from two hospitals had received treatment under the same treatment protocol and dose adjustment guidelines to maintain the ANC levels within target levels (500–1,500/µL). Genotype-guided dose modification was not conducted. Additional demographic data are shown in **Table 1**.

**Table 1:** Clinical characteristics of study subjects.

Characteristics	Study cohorts		
	AMC	SNUH	Total
**No. of subjects**	95	149	244
**Age at diagnosis (year)**, mean ± SD^†^	5.23 ± 1.8	8.57 ± 4.6	7.26 ± 4.1
**Sex**			
Male	52	93	145
Female	43	56	99
**Last-cycle 6-MP dose (mg/m^2^/day),** mean ± SD (*N*)			
6-MP < 12.5	8.14 ± 1.7 (2)	6.25 ± 2.9 (4)	6.88 ± 2.6 (6)
12.5 ≤ 6-MP < 25	17.39 ± 3.4 (4)	19.40 ± 3.6 (9)	18.78 ± 3.7 (13)
25 ≤ 6-MP < 37.5	32.19 ± 3.4 (10)	30.72 ± 4.0 (16)	31.28 ± 3.8 (26)
37.5 ≤ 6-MP < 50	44.52 ± 3.7 (13)	45.80 ± 3.5 (14)	45.18 ± 3.6 (27)
6-MP ≥ 50	79.15 ± 18.1 (66)	78.84 ± 23.1 (106)	78.96 ± 21.3 (172)
Total	65.37 ± 26.6 (95)	65.03 ± 30.0 (95)	65.16 ± 28.7 (244)

^†^Data for age at diagnosis were not available for one subject. 6-MP, 6-mercaptopurine; AMC, Asan Medical Center; SNUH, Seoul National University Hospital.

### Data Generation and Sequencing

Exome sequencing was performed using Ion AmpliSeq^™^ Exome panel to screen coding sequence region of entire genome. This panel included the exome of 19,072 genes and the size of the total targeted region was 57.7 Mb. The panel contained 293,903 primer pairs that were multiplexed into 12 pools to avoid primer-dimer formation and interference during PCR. The range of amplicons amplified by these oligo primer pairs ranged from 125 to 275 bp, and the rate of “on target” coverage for this panel was 95.69%. PCR assays were performed directly to amplify 100 ng of genomic DNA samples extracted from normal blood cells in bone marrow aspirates or peripheral blood so as to collect the target regions using the oligo primer pairs of the panel. Reaction parameters were as follows: 99°C for 2 min, followed by 10 cycles of 99°C for 15 s, 60°C for 16 min, and 10°C for 1 min. After amplification, library construction was performed by using the Ion AmpliSeq library kit plus as described in the manufacturer’s instructions (Thermo Scientific, Waltham, MA). Libraries were quantified using an Agilent 2100 Bioanalyzer (Agilent, Santa Clara, CA) and then diluted to ∼10 pM. Subsequently, 33.3 μL of the barcoded libraries was combined in sets of three barcodes. The combined libraries were sequenced using the Ion Proton platform with PI chip V3, following the manufacturer’s instructions (Thermo Scientific, Waltham, MA). Reads were mapped to the human reference genome build (hg19) with a mapping alignment program from Thermo Fisher (version 4.4, Torrent Suite Software) on germ-line and low stringency settings (minimum observed allele frequency required for a non-reference variant call is 0.18 for single-nucleotide variant (SNV) and 0.23 for InDel, minimum phred scales call quality is 14 for SNV and 19 for InDel, minimum coverage for called variants is 35 for SNV and 40 for InDel, and maximum strand bias is 0.95 for SNV and 0.75 for InDel). Single-nucleotide variants (SNVs) and short insertions/deletions (InDels) were identified *via* Genome Analysis Toolkit (GATK) 2.8-1 Unified Genotyper (DePristo et al., 2011). To estimate the pathogenicity of variants, two *in silico* variant deleteriousness prediction scores were annotated: sorting intolerant from tolerant (SIFT) (Ng, 2003) and combined annotation dependent depletion (CADD) (Kircher et al., 2014). The protein-coding gene region was defined using ANNOVAR (http://annovar.openbioinformatics.org/) (Wang et al., 2010). All the variants identified in 244 ALL samples are described in **Supplementary Table S1** and **S2**.

### Calculation of Gene-Wise Variant Burden Score

Gene-wise deleterious variant burden was computed for *NUDT15* and *TPMT* as described by Lee et al. (2016) and Seo et al. (2018). Under the hypothesis that variants that have potential effects to change protein function not necessarily guarantee but have power to cause harmful phenotypes, only variants with SIFT scores less than 0.7 were further considered.

$$G_{i}=\{v|v\text{ with a SIFT score less than }0.7\}$$

As SIFT does not provide functional scores for InDels, _adj_ *v* for all InDel variants were assigned as 1e-8 under the hypothesis that InDels are more deleterious than single-nucleotide substitutions. Considering the dosage effects, adjusted SIFT score_ adj_ *v* was calculated for each variant according to their genotype.

$${}_{\text{adj}}v_{j}=\{\begin{array}{ll} {\left(\text{SIFT score}\right)}^{0.5} & ,\text{ if }v_{j}\in G_{i}\text{ and heterozygote}\\ \text{SIFT score} & ,\text{ if }v_{j}\in G_{i}\text{ and homozygote} \end{array}$$

For each gene *G* *_i_* with *n* deleterious variants, we calculated GVB(*G* *_i_*), the cumulative genic effect for all coding variants of the gene, by calculating the geometric mean of _adj_ *v* (Equation). GVB_g_ is considered as 1 if the count *n* of variant *j* with scores less than 0.7 in a gene is 0, indicating that the gene is not displaying any deleterious variant.

$$\text{GVB}(G_{i})=\{\begin{array}{cc} 1 & ,\text{if }n(G_{i})=0\\ {\left(\prod_{j=1}^{n}{}_{\mathrm{adj}}v_{j}\right)}^{\frac{1}{n}} & ,\text{if }n(G_{i})>0 \end{array}$$

We obtained GVB_g_ values for each individual ranging from 0 to 1. To predict 6-MP sensitivity, GVB*^NUDT15^* ^,^ *^TPMT^* was generated by calculating the geometric mean of GVB*^NUDT15^* and GVB*^TPMT^*.

$${\text{GVB}}^{\mathrm{NUDT}15,\mathrm{TPMT}}={\left({\text{GVB}}^{\mathrm{NUDT}15}\times {\text{GVB}}^{\mathrm{TPMT}}\right)}^{\frac{1}{2}}$$

### Prediction of Star Allele Diplotypes for 244 Acute Lymphoblastic Leukemia Samples

To classify 244 ALL samples into three metabolizer groups, we inferred haplotypes using the PHASE 2.1.1 software (Stephens et al., 2001; Stephens and Scheet, 2005) (**Supplementary Figure S3**). On the basis of the inferred haplotype information, we extracted star alleles that matched the haplotype set translational table from PharmGKB (https://www.pharmgkb.org/) (Whirl-Carrillo et al., 2012). Predicted genotypes were translated into molecular phenotypes on the basis of the coded genotype–phenotype translation tables from Moriyama et al. (2016) for *NUDT15* and from PharmGKB tables for *TPMT*.

### Estimation of Diagnostic Accuracies by Receiver Operating Curve Analyses

To assess prediction accuracies, we calculated DIP, the percentage of the actual administered dose to the planned dose, as an index for 6-MP drug toxicity. Dose in the last maintenance cycle was used, since the doses of 6-MP in the final maintenance cycle were supposed to be the maximum tolerated doses for patients (Kim et al., 2012). DIP prediction accuracies of GVB (GVB*^NUDT15^*, GVB*^TPMT^*, and GVB*^NUDT15^* ^,^ *^TPMT^*) and star allele-based predictions were compared using AUROC analysis with the R language pROC package (Robin et al., 2011). We computed specificity, sensitivity, PPV, and NPV under the binary classification model with nine different cutoff levels (i.e., 5%, 10%, 15%, 25%, 35%, 45%, 60%, 80%, and 100%) for defining high-risk DIP groups. All statistical analyses were performed using R version 3.5.1.

## Results

### Relation of Gene-Wise Variant Burden and Star Allele-Based Molecular Phenotypes

*NUDT15* and *TPMT* haplotypes of each subject were first inferred from whole exome sequencing (WXS) data by using the PHASE tool, and matched star allele genotypes were assigned for each subject. The star allele genotypes were then translated into three molecular phenotype groups according to their allele combinations; poor (PM, No function|No function), intermediate (IM, Normal|No function or Normal|Decreased), and normal (NM, Normal|Normal) metabolizers. Six and four star alleles were identified for *NUDT15* and *TPMT* genes, respectively, from the 244 ALL patients with their frequencies (**Table 2**). **Table 3** shows the distribution of subsequently predicted enzymatic metabolizer phenotypes for *NUDT15* and *TPMT* among the 244 ALL patients.

**Table 2:** Alleles identified in 244 ALL samples with known allele functions.

Gene	Number of identified alleles	Alleles identified in 244 ALL samples	Frequencies (%)
*NUDT15*	6	*1	438 (89.75)
		*2	6 (1.23)
		*3	35 (7.17)
		*4	4 (0.82)
		*5	4 (0.82)
		*6	1 (0.20)
*TPMT*	4	*1	127 (26.02)
		*1S	354 (72.54)
		*3C	6 (1.23)
		*6	1 (0.20)

Haplotypes were inferred via PHASE 2. Star alleles were assigned by the PharmGKB haplotype set translational table. ALL, acute lymphoblastic leukemia.

**Table 3:** Distribution of predicted enzymatic metabolizer phenotypes.

Molecular phenotype	Function	*NUDT15*	*TPMT*
Poor (%)	No function | No function	1 (0.41)	NA
Intermediate (%)	Normal | No function	48 (19.67)	6 (2.46)
	Normal | Decreased	NA	1 (0.41)
Normal (%)	Normal | Normal	195 (79.92)	237 (97.13)
Total (%)		244 (100)	244 (100)

Molecular phenotypes were assigned using the PharmGKB haplotype set translational table. Star (*) allele genotype-to-phenotype correlation was adapted from information available at the Moriyama et al. (NUDT15) and the Clinical Pharmacogenetics Implementation Consortium (CPIC) guideline ( TPMT ); NA, not available.

While 49 (20.1%) of 244 ALL patients were classified into non-NM (one PM and 48 IMs) phenotype for *NUDT15*, only seven (2.9%) IMs were identified for *TPMT*, reflecting ethnic variation of *NUDT15* and *TPMT* variants, in a consistent manner (**Table 3**). Since individuals with *TPMT* homozygous mutant alleles are rarely observed in East Asian population, none of the patients were classified into the poor metabolizer group. IMs were stratified into two groups: 1) individuals carrying one copy of a normal function allele and one copy of a *decreased function* allele and 2) individuals carrying one copy of a normal function allele and one copy of *no function* allele. Carriers of non-functional allele, compared with carriers of decreased function allele, are considered to be at an increased risk for functional decline.

**Figure 1:**
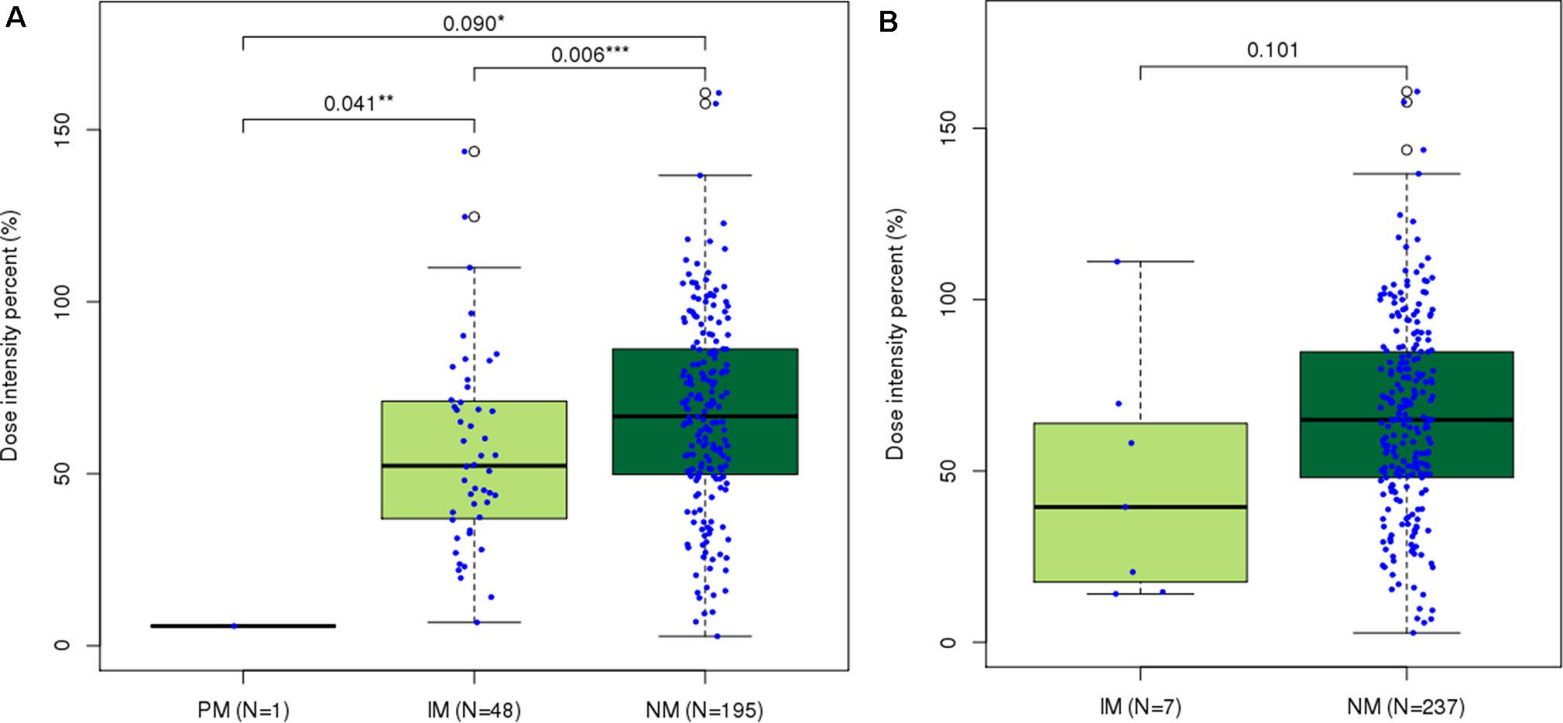
Distribution of last-cycle dose intensity percent of 6-mercaptopurine according to star allele-based molecular phenotype groups in ALL. Dose intensity percent distribution across **(A)** *Nudix Hydrolase 15 (NUDT15)* and **(B)** *Thiopurine S-Methyltransferase (TPMT)* molecular phenotype groups. Normal metabolizers of *NUDT15* showed significantly higher dose intensity percent than did intermediate ( *p* = 0.006) and poor ( *p* = 0.090) metabolizers. *p < 0.1, **p < 0.05, and ***p < 0.01 by Mann–Whitney *U* test.

Patients with *NUDT15* normal metabolizing alleles (DIP = 67.608 ± 28.2, *n* = 195) tolerated significantly higher DIPs of 6-MP than did slow metabolizers [5.712 (PM, *n* = 1), 56.452 ± 28.2 (IM, *n* = 48)] (**Figure 1A**). Clinical usefulness of the conventional star allele-based classification was successfully demonstrated for *NUDT15* variants in the present study. Due to the small number of non-NM subjects for *TPMT* in Korean ALL patients, the difference of DIPs between NM (65.702 ± 28.4, *n* = 237) and IM (46.805 ± 35.7, *n* = 7) did not reach statistical significance (*p* = 0.10, **Figure 1B**).

**Figure 2:**
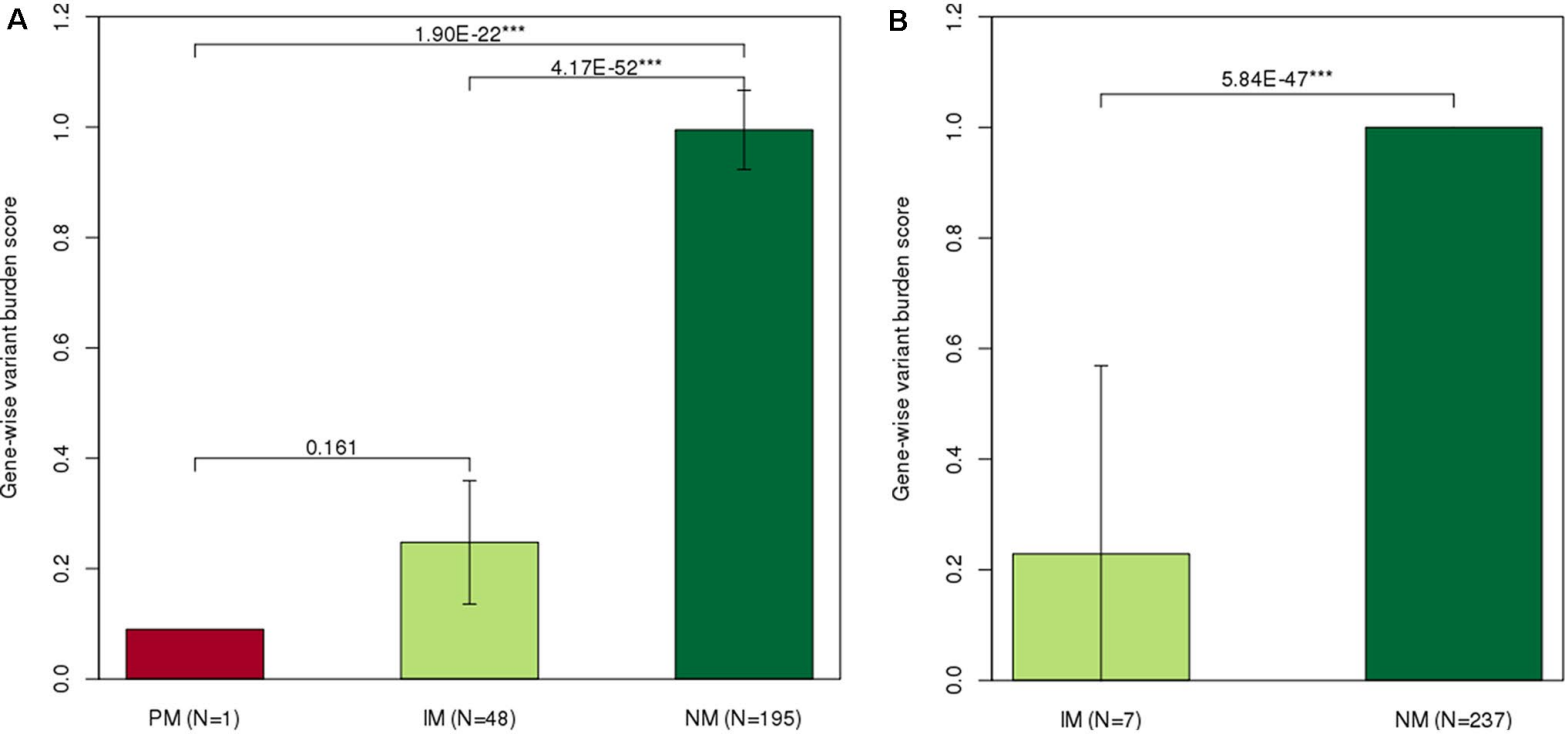
Distribution of gene-wise variant burden (GVB) scores according to the star allele-based molecular phenotype groups. Gene-wise variant burden (GVB) scores across **(A)** *NUDT15* and **(B)** *TPMT* molecular phenotype groups. Normal metabolizers showed significantly higher dose intensity percent than did intermediate (*NUDT15*, *p* = 4.17E−52; *TPMT* *p* = 5.84E−47) and poor (*NUDT15*, *p* = 1.9E−22) metabolizers. *p < 0.1, **p < 0.05, and ***p < 0.01 by Mann–Whitney *U* test.

GVB scores among different molecular phenotype groups for *NUDT15* (PM = 0.09, IM = 0.248 ± 0.1, and NM = 0.995 ± 0.1, **Figure 2A**) and for *TPMT* (IM = 0.229 ± 0.3, NM = 1 ± 0.0, **Figure 2B**) showed statistically significant differences. The observed positive correlation between our GVB score and the conventional enzymatic metabolizer phenotypes for both *NUDT15* and *TPMT* variants strongly supported our further analysis. Note that those pharmacogenetic star alleles have long been empirically developed by clinical case–control studies and/or animal and molecular studies. In contrast, the GVB analysis is based on purely theoretical *ab initio* and *in silico* methods without requiring empirical studies that are prohibitively costly considering the numerous drugs and genetic variants discovered by NGS technologies and the interactions. In the following sections, we explore the potential of the GVB scoring method for predicting DIPs as an indicator of 6-MP intolerance in pediatric ALL patients.

### Gene-Wise Variant Burden Scores for Predicting Last-Cycle 6-Mercaptopurine Dose Intensity Percent

**Figure 3:**
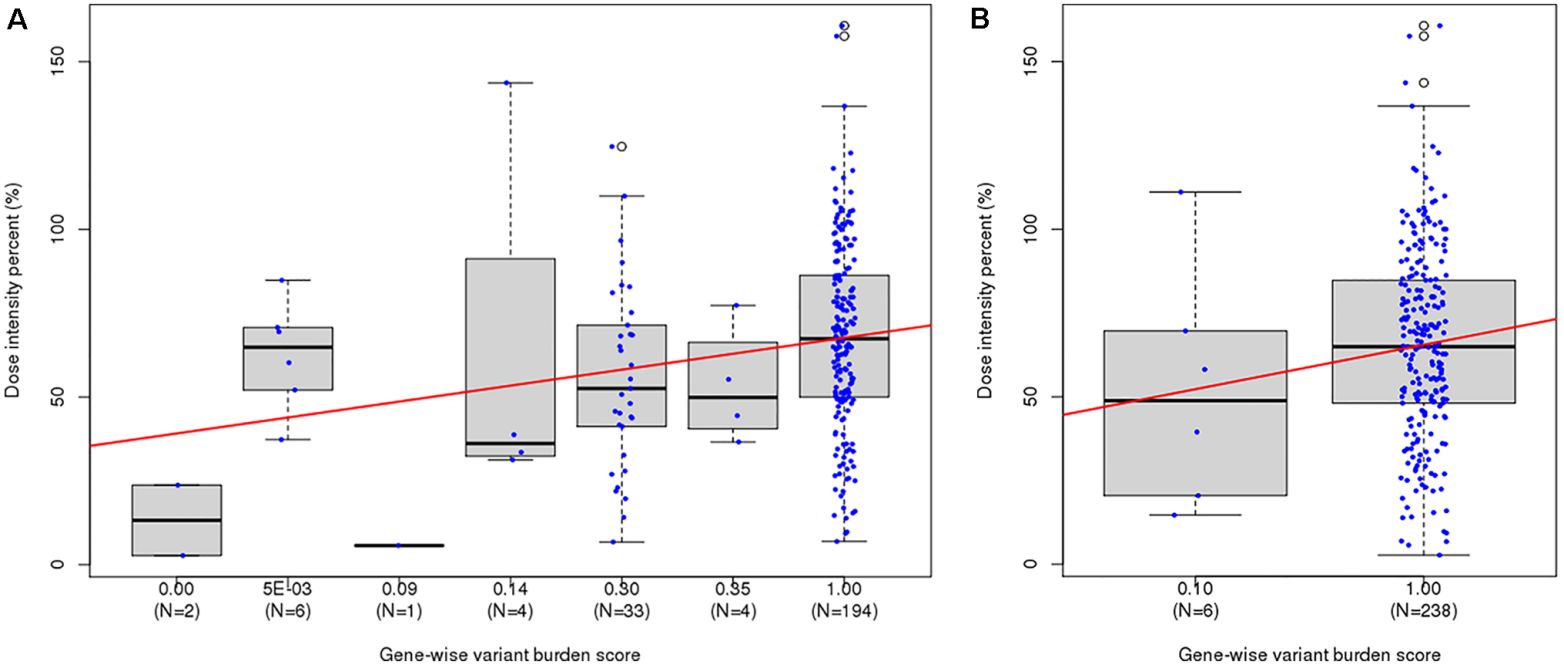
Distribution of last-cycle dose intensity percent of 6-mercaptopurine according to gene-wise variant burden (GVB) score bins. **(A)** GVB*^NUDT15^* [Kruskal–Wallis *p*-value = 0.016, Spearman’s rank correlation *p*-value = 0.001 ( *ρ* = 0.21), and Kendall’s rank correlation *p*-value = 0.001 (*τ* = 0.17)]. **(B)** GVB*^TPMT^* [Kruskal–Wallis *p*-value = 0.271, Spearman’s rank correlation *p*-value = 0.272 ( *ρ* = 0.07), and Kendall’s rank correlation *p*-value = 0.271 (*τ* = 0.06)].

Since both *NUDT15* and *TPMT* genes are not highly variable, only seven and two GVB value groups for *NUDT15* and *TPMT*, respectively, were identified in the 244 ALL patients. GVB*^NUDT15^* demonstrated statistically significant positive correlation with DIP (*p* = 0.016 by Kruskal–Wallis test, *p* = 0.001 (*p* = 0.21) by Spearman’s rank correlation, *p* = 0.001 (*τ* = 0.17) by Kendall’s rank correlation) (**Figure 3A**). Exclusion of the two patients having both *NUDT15* and *TPMT* variants slightly improved statistical significance (**Supplementary Figure S4**). Due to the low frequency of *TPMT* alleles in East Asian population, 97.5% (*n* = 238) of all ALL patients were classified into wild type (GVB*^TPMT^* = 1.00 ± 0.00) and only six (2.50%) were classified into variant type (GVB*^TPMT^* = 0.10 ± 0.00) groups, resulting in poor statistical significance (*p* = 0.408 by *T*-test, *p* = 0.272 (*ρ* = 0.07) by Spearman’s rank correlation, *p* = 0.271 (*τ* = 0.06) by Kendall’s rank correlation) (**Figure 3B**).

### Performance Comparisons Between Gene-Wise Variant Burden and Star Allele-Based Molecular Phenotypes Across Different Risk Group Decision Thresholds

**Figure 4:**
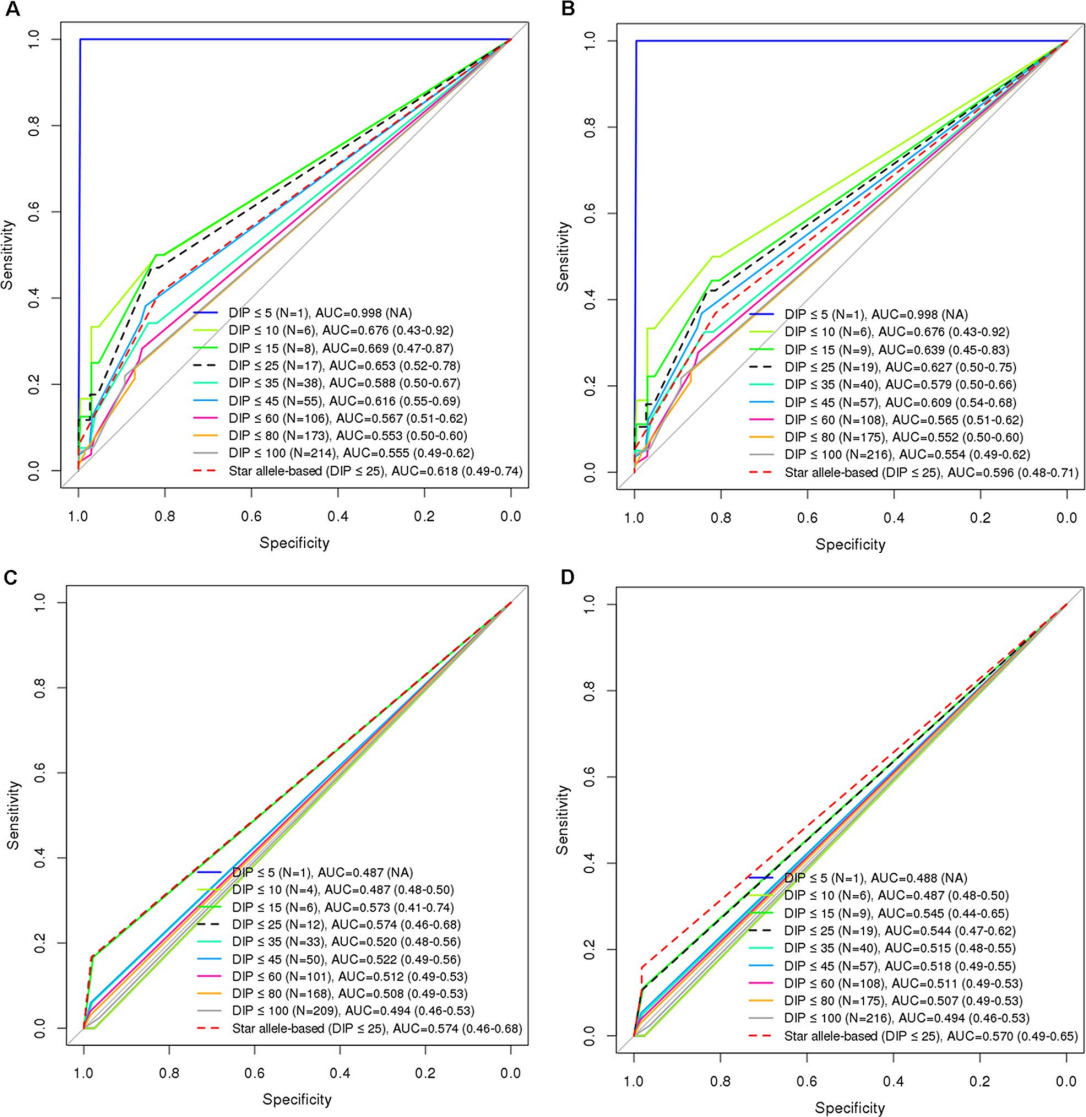
Comparison of diagnostic accuracies between star allele-based molecular phenotyping and GVB scoring for 6-mercaptopurine intolerance in ALL. Diagnostic accuracies are measured by using AUROC analysis for **(A)** GVB*^NUDT15^* excluding two subjects with *TPMT* variants (DeLong’s *p*-value = 0.163), **(B)** GVB*^NUDT15^* (DeLong’s *p*-value = 0.163), **(C)** GVB*^TPMT^* excluding seven subjects with *NUDT15* variants (DeLong’s *p*-value = 0.5), and **(D)** GVB*^TPMT^* (DeLong’s *p*-value = 0.841). Numbers in the last parentheses indicate area under the curve (AUC) with 95% confidence intervals. DIP, dose intensity percent; AUC, area under the curve.

Using ROC analysis, we evaluated the performances of GVB at nine cutoff levels (i.e., DIP < 5%, 10%, 15%, 25%, 35%, 45%, 60%, 80%, and 100%) for defining the 6-MP high-risk groups. Star allele-based classification was also applied for systematic comparison across different DIP threshold levels. DIP below 25% of planned dose of 6-MP is a generally accepted threshold for predicting 6-MP intolerance. **Figure 4A** demonstrates that GVB*^NUDT15^* showed better AUCs at all threshold DIP levels below 25% (0.998 (DIP < 5%), 0.676 (DIP < 10%), 0.669 (DIP < 15%), and 0.653 (DIP < 25%)) than did the conventional star allele-based molecular phenotypes (AUC = 0.618). Moreover, exclusion of the two confounding patients with both *NUDT15* and *TPMT* variant alleles slightly improved performances than did both before-exclusion GVB*^NUDT15^* at all threshold DIP levels below 25% [AUC = 0.998 (DIP < 5%), 0.676 (DIP < 10%), 0.639 (DIP < 15%), and 0.627 (DIP < 25%)] and the star allele-based (AUC = 0.596) analyses (**Figure 4B**). Mainly due to the low frequency of *TPMT* variant alleles in East Asian population, both GVB*^TPMT^* and star allele-based predictions using *TPMT* seem to show poor AUCs for predicting DIP at all threshold levels (**Figure 4C and D**).

**Figure 5:**
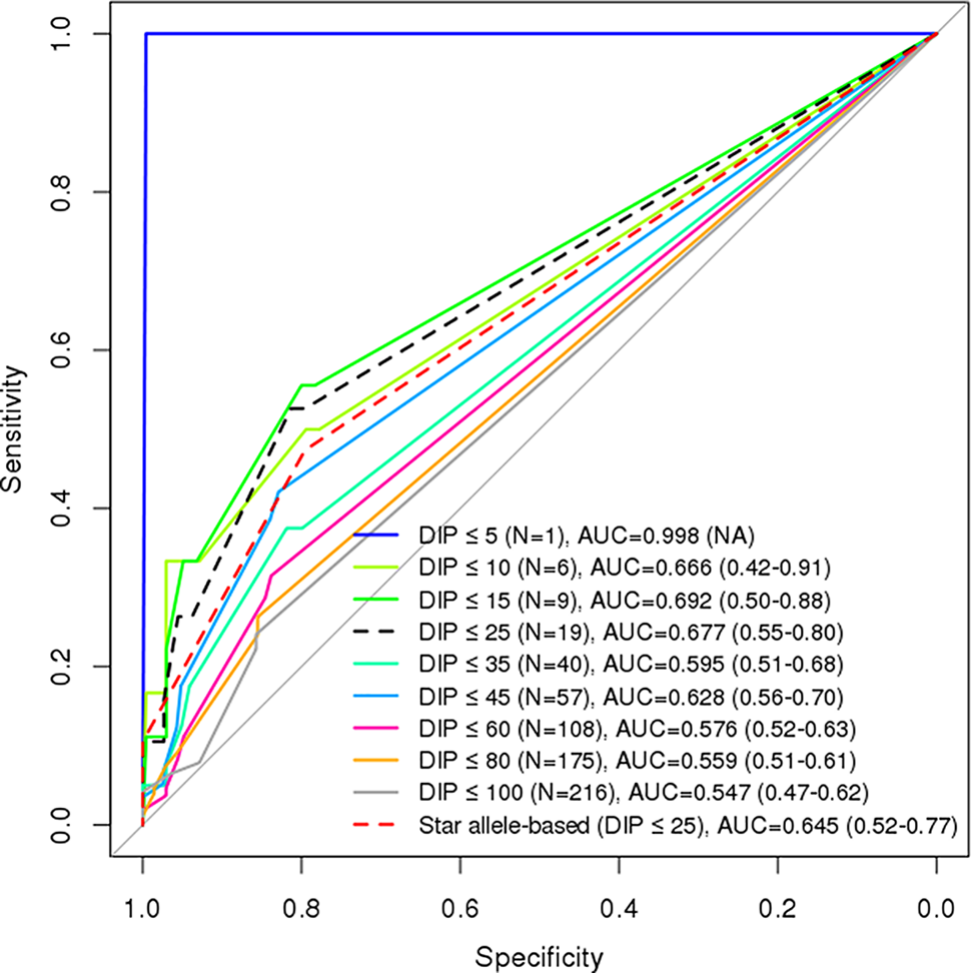
Comparison of diagnostic accuracies between combined (*NUDT15* and *TPMT*) star allele-based molecular phenotyping and GVB scoring for 6-mercaptopurine intolerance in ALL. Diagnostic accuracies are measured by using AUC analysis for GVB*^NUDT15^* ^,^ *^TPMT^* (DeLong’s *p*-value = 0.175). Numbers in the last parentheses indicate AUC with 95% confidence intervals. DIP, dose intensity percent; AUC, area under the curve.

More importantly, we performed ROC analysis by aggregating the genetic effects of these two genes, *NUDT15* for East Asian and *TPMT* for European heritages. We computed and evaluated GVB*^NUDT15^* ^,^ *^TPMT^*, which outperformed GVB*^NUDT15^* or GVB*^TPMT^* alone as well as the combined molecular phenotypes of both *NUDT15* and *TPMT* at all DIP threshold levels (**Figure 5**). In summary, at the clinically important DIP level of below or above 25%, the best AUC values for GVB*^NUDT15^* ^,^ *^TPMT^*, GVB*^NUDT15^*, GVB*^TPMT^*, and combined star alleles were 0.677, 0.653, 0.574, and 0.645, respectively. GVB*^NUDT15^* ^,^ *^TPMT^* not only showed the best performance but also successfully included the two confounding patients with both *NUDT15* and *TPMT* variant alleles. While combining GVB scores of multiple genes is simple and straightforward, it is not the case for star alleles, which do not provide a uniform way of combining method for multiple genes.

### Comparison of Prediction Accuracies Between Gene-Wise Variant Burden and Star Allele-Based Methods

To test the clinical utility of GVB method for guiding 6-MP dosing and/or for providing systematic framework for clinical studies of 6-MP intolerance and its genetic determinants of *NUDT15* and *TPMT* for predicting DIP groups, we evaluated the diagnostic characteristics of the conventional star allele-based and GVB scoring methods in a simulated clinical setting. **Table 4A and 4B** exhibits diagnostic accuracies for star allele-based molecular phenotype groups and gene-wise variant burden score groups, respectively, for 6-MP intolerance among 244 pediatric ALL patients by the last-cycle DIP of 6-MP. Of the 244 ALL patients, 189 (84.4%) exhibited no *NUDT15* or *TPMT* variant and hence was classified into NMs for both genes (**Table 4A**). Of the rest 55 non-NM patients, nine (16.4%) showed DIP below 25%, while 10 of 189 (5.3%) NM patients showed low DIP values.

**Table 4:** Comparison of star allele-based haplotyping versus gene-wise variant burden (GVB*^NUDT15^* ^,^ *^TPMT^*) analyses for 6-mercaptopurine intolerance measured by last-cycle dose intensity percent in ALL. Diagnostic accuracy table of **(A)** star allele-based haplotypes and dose intensity percent groups and **(B)** gene-wise variant burden score and dose intensity percent groups.

(A)				
*NUDT15 and TPMT metabolizer*	Dose intensity percent groups		Total	
	≤25	>25		
**PM + IM**	9	46	55	PPV 16.36% (9/55)
**NM**	10	179	189	NPV 94.70% (179/189)
**Total**	19	225	244	
	Sensitivity 47.36% (9/19)	Specificity 79.56% (179/225)		Accuracy 77.05% (188/244)
(B)				
*Gene-wise variant burden score*	Dose intensity percent groups		Total	
	≤25	>25		
GVB*^NUDT15,TPMT^***≤ 0.3**	10	42	52	PPV 19.23% (10/52)
GVB*^NUDT15,TPMT^* **> 0.3**	9	183	192	NPV 95.31% (183/192)
**Total**	19	225	224	
	Sensitivity 52.63% (10/19)	Specificity 81.33% (183/225)		Accuracy 79.10% (193/244)

PM, poor metabolizer; IM, intermediate metabolizer; NM, normal metabolizer; PPV, positive predictive value; NPV, negative predictive value.

Although one can choose many threshold levels of GVB, because star alleles can just provide a small number of categories, we chose the most reliable binning threshold of GVB*^NUDT15^* ^,^ *^TPMT^* ≤ 0.3, the cut-point that maximizes the Youden index (**Supplementary Figure S5**), for classifying the patients into the below and above 25% DIP groups as shown in **Table 4B**. It is a coincidence that Lee et al. (2016) also suggested GVB*^Pharmacogenes^* ≤ 0.3 as the threshold for predicting pharmaceutical market withdrawals in general. GVB*^NUDT15^* ^,^ *^TPMT^* correctly classified one more high-risk (DIP ≤ 25%) and four more low-risk (DIP > 25%) patients into the correct-risk groups (**Table 4B**) than did the traditional haplotype-based method (**Table 4A**), with an improved sensitivity from 47.36% to 52.63% and an improved specificity from 79.56% to 81.33%, though the difference did not reach statistical significance (*p*-value for sensitivity = 1 and *p*-value for specificity = 0.134, as determined using a McNemar test). Both PPV and NPV increase from 16.36% to 19.23% and from 94.70% to 95.31%, respectively. Overall, it is suggested that the “computational” GVB*^NUDT15^* ^,^ *^TPMT^* is an improved or at least comparable predictor than the “empirical” star allele-based haplotypes for determining subjects with increased risk of 6-MP intolerance in pediatric ALL patients measured by the last-cycle 6-MP DIP.

## Discussion

An enduring challenge in precision medicine is to predict adequate drug responses for individual patients (Shah and Shah, 2012). Recent discoveries have revealed a few highly functional and clinically relevant novel variants associated with 6-MP intolerance. However, since implicating drug toxicity based on a single variant is notoriously unreliable as shown in **Supplementary Figure S1** for SIFT and **Supplementary Figure S2** for CADD, developing strategies to aggregate the key effects over a range of genomic region is highly required. In the present study, we evaluated the utility of gene-wise deleterious variant burden scoring method, as a sequencing-based, simple, reliable, quantitative, and easy-to-compare score for predicting 6-MP intolerance of 244 pediatric ALL patients. In addition to DIP, GVB showed a statistically significant negative correlation with the incidence of grade 4 neutropenia (*p* = 1.43E−04 by Kruskal–Wallis test, *p* = 3.89E−07 (*ρ* = −0.32) by Spearman’s rank correlation, and *p* = 8.06E−07 (*τ* = −0.27) by Kendall’s rank correlation (**Supplementary Figure S6**). This implies that GVB is a reliable score that can predict hematological toxicity in pediatric ALL patients. When beginning treatment, NGS-based drug intolerance prediction is useful because it is practical to detect patients at high risk of toxicity. For example, patients with low GVB have a high probability of 6-MP toxicity at the initial recommended dose range; thus, clinicians may attempt to reduce the initial target dose of 6-MP. After an initial target dose is determined, a close therapeutic drug monitoring could help to avoid potential causes for toxicity, such as clinically relevant drug–drug interactions, reduced drug clearance due to liver and/or renal impairment, and altered drug utilization due to physiological conditions, as a complementary type of practice during the treatment (Ju-Seop Kang, 2009).

GVB analysis has several benefits over conventional star allele-based approaches. GVB 1) quantitates gene-wise variant burden with a single score; 2) provides a measure of inter-individual genetic variability for each gene; 3) considers common, rare, and novel genetic variants together; 4) provides an ethnic variability-neutral method for studying pharmacogenomics; 5) provides a systematic and reliable framework for designing further pharmacogenomics studies considering many gene interactions for clinical endpoints; and 6) adopts the contributing effect of novel low-frequency variants with potentially reduced function in predicting individual drug toxicity.

Based upon the very recent CPIC updates on *NUDT15*, three newly enrolled alleles were characterized (Moriyama et al., 2017). Since new haplotype designation is highly dependent on the characteristics of the study population, there will be restrictions in incorporating new or even as-yet-unidentified evidences in predicting future drug intensity. GVB can be used to develop a model to determine optimal doses without requiring a multi-ethnic population study, especially for under-studied subpopulations.

The following limitations are inherent in the present study. To evaluate the validity of GVB, independent replication studies for an expanded gene–drug set with sufficient sample sizes in diverse ethnic groups are required as no novel variant was identified in the current study. A conventional single variant-based association test of rare variants requires infeasible magnitude of sample sizes (Bansal et al., 2010), but approaches that aggregate common, rare, and novel variants jointly will substantially reduce a required effective sample sizes (Witte, 2012). The robustness of the analysis framework shall further be improved as novel prognostic markers on 6-MP DIP are acquired. The limitations in interpreting the score includes that all InDels are treated as highly damaging as SIFT provides scores for only single-nucleotide variants. As there are many *in silico* variant deleteriousness scoring method based on different principles, comprehensive evaluation of different method is required (**Supplementary Figure S7**). We also performed CADD-based computation of GVB values, resulting in similar results (**Supplementary Figures S8 and S9**). It has been reported that CADD tends to evaluate in-frame InDels as relatively benign (Kircher et al., 2014). However, recent *in vitro* activity assay of *NUDT15* (Moriyama et al., 2017) proved that in-frame InDel carriers are more likely to be in states with severely diminished response to 6-MP. It is strongly recommended that for clinical applications, potential clinical impacts of genetic variants on drug sensitivity should be further examined to improve estimation accuracy, as *in silico* prediction scores can exhibit incorrect predictions. Producing a custom capture panel for clinically actionable genes could be more cost-effective than an exome-based approach.

One subject who was correctly classified by GVB carried a low-frequency novel deletion and predicted to belong to the high-risk group by GVB, whereas star allele-based prediction classified this patient into the NM group for both *NUDT15* and *TPMT*. The patient required reduced dose than recommended (DIP = 23.7%), supporting that GVB analysis resulted in 6-MP dose-related adverse drug reactions. The patient’s variant was heterozygous p.Gly17_Val18del, which was very recently assigned as NUDT15*9 with uncertain functionality. The other four who were correctly classified by GVB had p.Arg139His on one allele, which has assigned them to the IM (NUDT15 *1/*4) group. GVB classified them as relatively safe for drug toxicity, and none of them required a 25% reduction from the starting dose. Additionally, one patient who was classified as high risk by GVB was assigned to IM for both *NUDT15* and *TPMT* and required a severely reduced dose (14%), suggesting that GVB*^NUDT15^* ^,^ *^TPMT^* exhibits benefits in aggregating effects of many moderate genetic determinants into a single quantitative value.

## Ethics Statement

The study was approved by the Asan Medical Center (AMC) Review Boards and the Institutional Review Board of Seoul National University Hospital (SNUH). Informed written consents for blood sampling and analyses were obtained from all participants.

## Author Contributions

YP and JK designed the model and the framework. HK, JC, HI, and HK collected samples and clinical data. BM and MS carried out the experiment. YP and SY analyzed the data and carried out the implementation. YP performed the calculations. YP and JK wrote the manuscript. JK conceived the study and was in charge of overall direction and planning. All authors read and approved the final manuscript.

## Funding

This research was supported by a grant (16183MFDS541) from the Ministry of Food and Drug Safety in 2019.

## Conflict of Interest Statement

The authors declare that the research was conducted in the absence of any commercial or financial relationships that could be construed as a potential conflict of interest.
