# Supplementary material for: Star Allele-Based Haplotyping versus Gene-Wise Variant Burden Scoring for Predicting 6-Mercaptopurine Intolerance in Pediatric Acute Lymphoblastic Leukemia Patients
Source: Front Pharmacol. 2019 Jun 11;10:654. doi: 10.3389/fphar.2019.00654 (PMC6580331; doi:10.3389/fphar.2019.00654)
Supplement: Supplementary file 1 [file Table_1.docx]

Supplementary Material

# Supplementary Figures and Tables

## Supplementary Figures


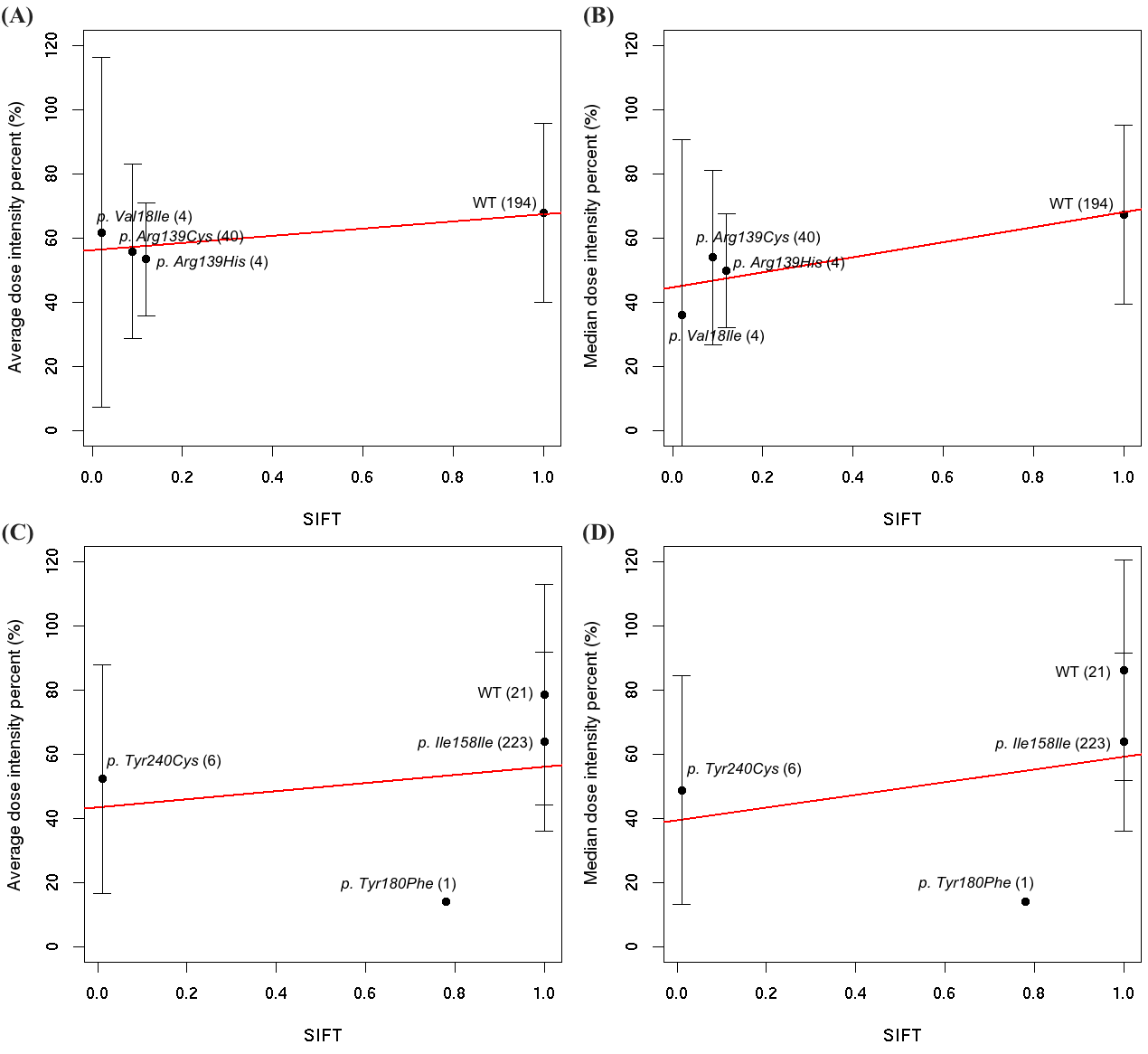


**Supplementary Figure S1. Scatter plots for comparing SIFT score (x-axis) to dose intensity percent (y-axis).** WT were considered as benign. (A) SIFT score for *NUDT15* variants vs. average dose intensity percent (*p*=0.917 (*ρ*=0.2) by Spearman’s rank correlation) (B) SIFT score for *NUDT15* variants vs. median dose intensity percent percent (*p*=0.333 (*ρ*=0.8) by Spearman’s rank correlation) (C) SIFT score for *TPMT* variants vs. average dose intensity percent percent (*p*=0.262 (*ρ*=0.7) by Spearman’s rank correlation) (D) SIFT score for *TPMT* variants vs. median dose intensity percent percent (*p*=0.262 (*ρ*=0.7) by Spearman’s rank correlation). Numbers in parentheses indicate number of samples.


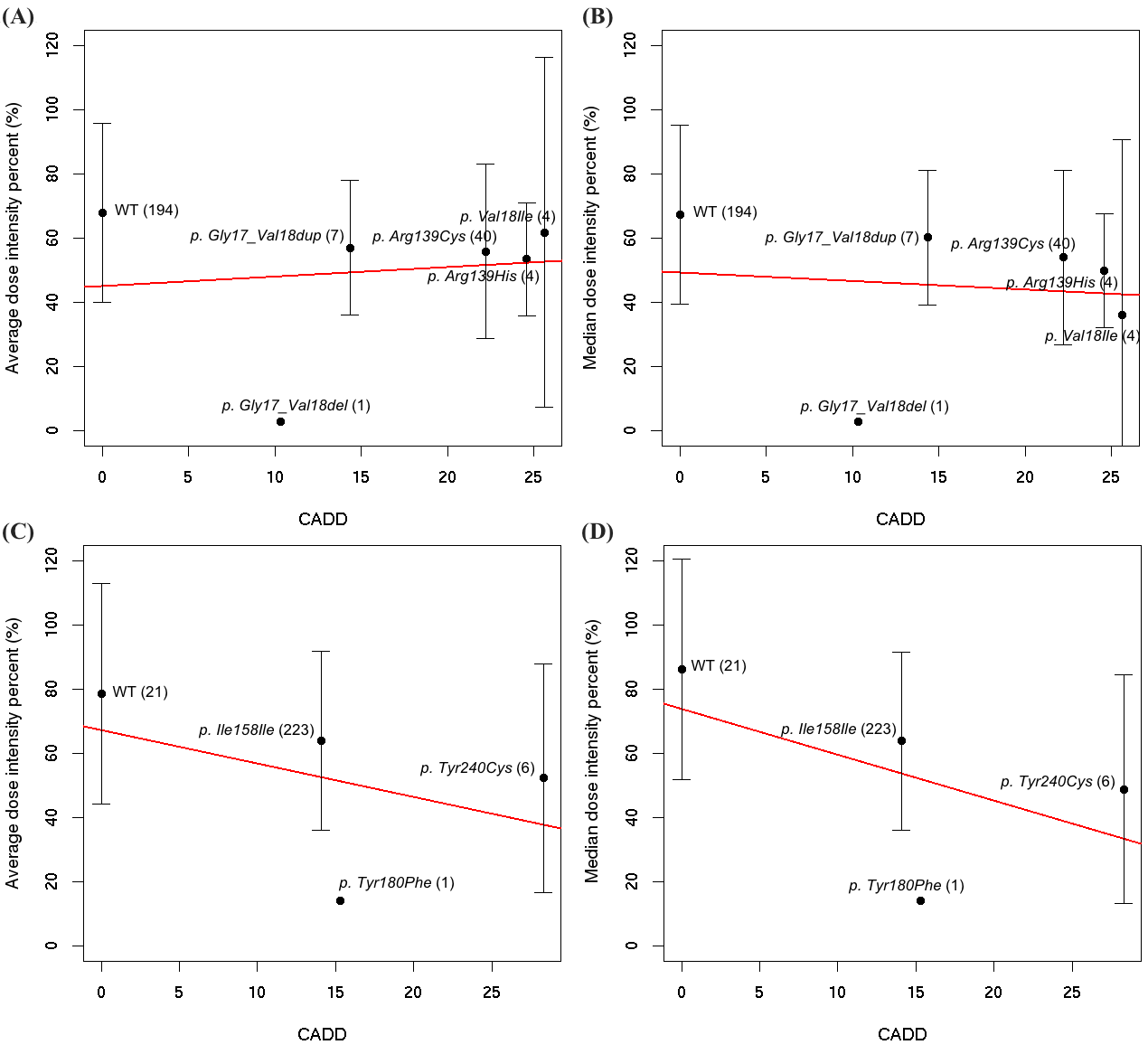
 **Supplementary Figure S2. Scatter plots for comparing CADD score (x-axis) to dose intensity percent (y-axis).** WT were considered as benign. (A) CADD score for *NUDT15* variants vs. average dose intensity percent percent (*p*=0.919 (*ρ*=-0.1) by Spearman’s rank correlation) (B) CADD score for *NUDT15* variants vs. median dose intensity percent percent (*p*=0.419 (*ρ*=-0.4) by Spearman’s rank correlation) (C) CADD score for *TPMT* variants vs. average dose intensity percent percent (*p*=0.333 (*ρ*=-0.8) by Spearman’s rank correlation) (D) CADD score for *TPMT* variants vs. median dose intensity percent (*p*=0.333 (*ρ*=-0.8) by Spearman’s rank correlation). Numbers in parentheses indicate number of samples.


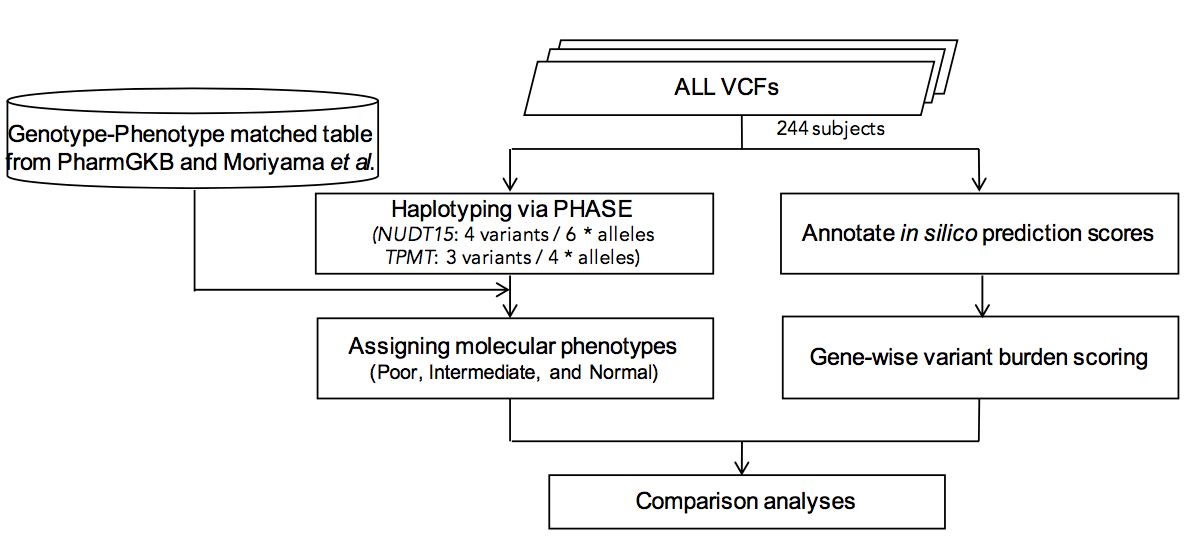


**Supplementary Figure S3. Schematic of the data analysis steps.** Individuals were classified as a Poor (PM), Intermediate (IM), or Normal (NM). PGS, personalized gene score.; ALL, Acute Lymphoblastic Leukemia


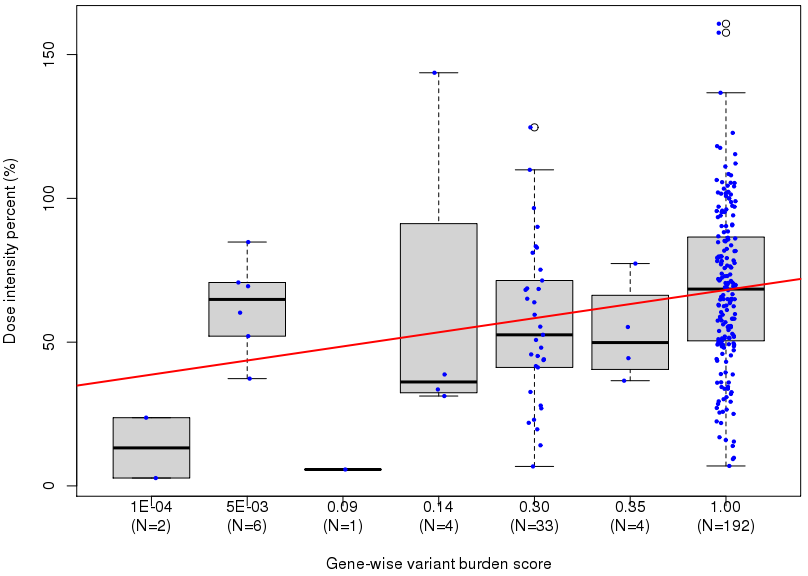
**Supplementary Figure S4. Distribution of dose intensity percent according to gene-wise variant burden scores excluding 2 subjects with *TPMT* variants.** (Kruskal-Wallis *p*-value = 0.012, Spearman’s rank correlation *p*-value = 7.2E-04 (ρ=0.22), Kendall’s rank correlation *p*-value=7.2E-04 (𝜏=0.17))


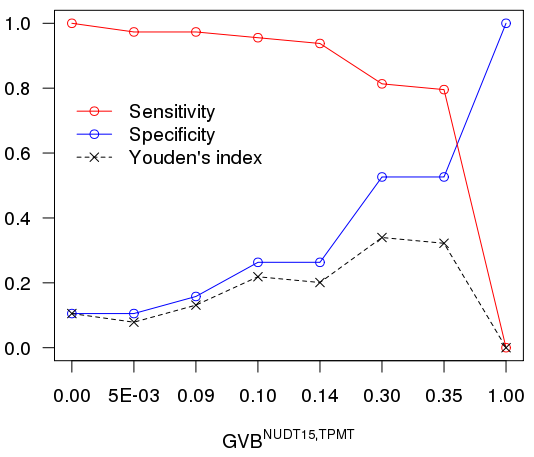


**Supplementary Figure S5. Sensitivity, specificity, and Youden's index for all GVB*^NUDT15,TPMT^* score values.** The cut-point which maximizes the Youden’s index means a reliable threshold.


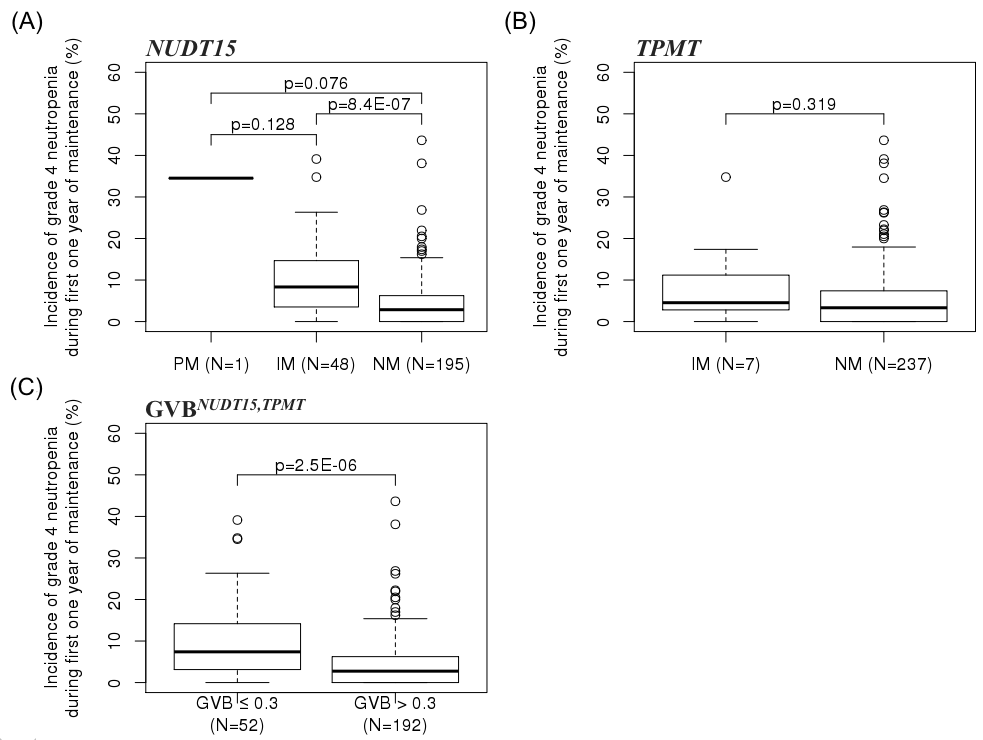


**Supplementary Figure S6. Incidence of Grade 4 neutropenia during first one year of maintenance according to gene-wise variant burden (GVB) score bins.** (A) star allele-based prediction for *NUDT15* vs. incidence of grade 4 neutropenia (B) star allele-based prediction for TPMT vs. incidence of grade 4 neutropenia (C) GVB*^NUDT15,TPMT^* vs. incidence of grade 4 neutropenia. Patients with low GVB*^NUDT15,TPMT^* had a low incidence of Grade 4 neutropenia. Statistical significance was calculated by Wilcoxon rank sum test; the incidence ratio of grade 4 neutropenia during first year of maintenance = the number of CBC tests with Grade 4 neutropenia / the total number of CBC tests performed


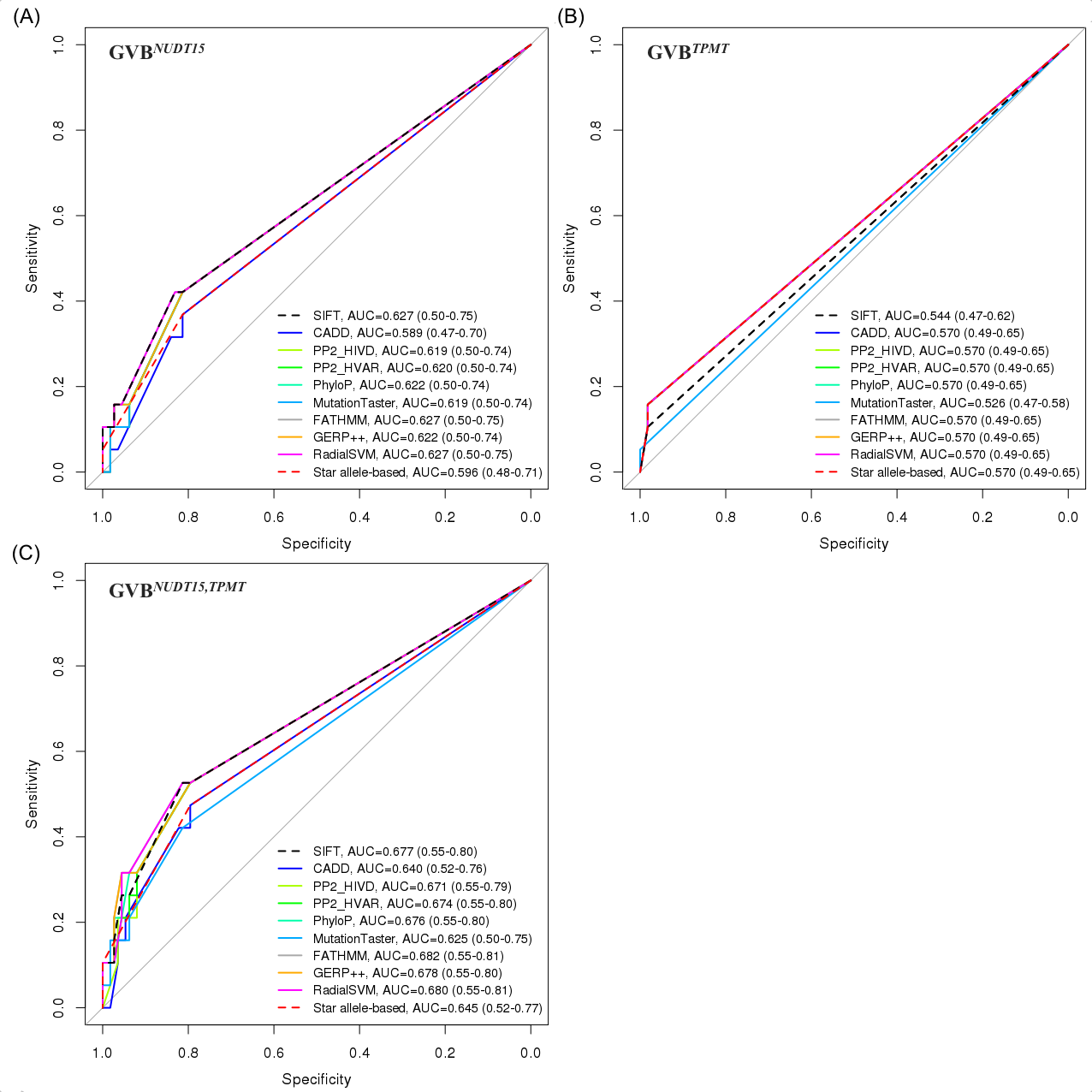
 **Supplementary Figure S7. Comparison of diagnostic accuracies between star allele-based molecular phenotyping and gene-wise variant burden scoring for 6-mercaptopurine intolerance in ALL using nine *in silico* prediction tools.** ROC curves for (A) GVB*^NUDT15^*, (B) GVB*^TPMT^*, and (C) GVB*^NUDT15,TPMT^*


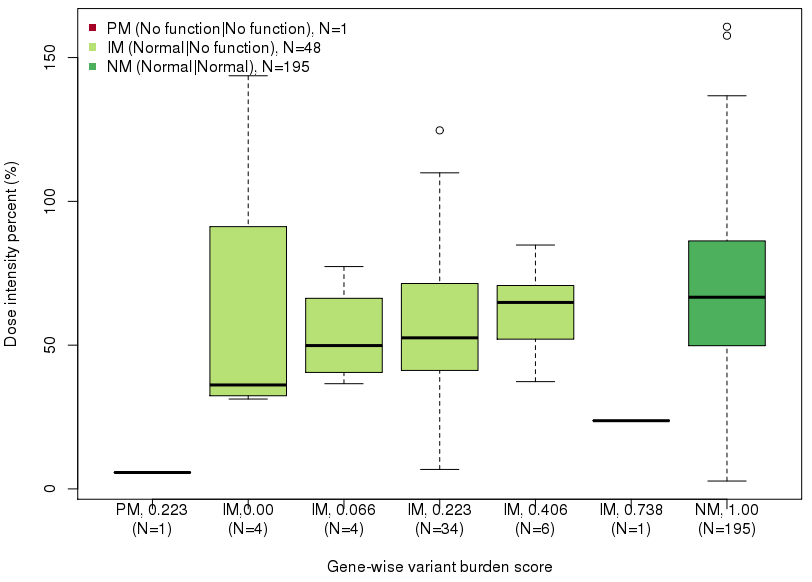


**Supplementary Figure S7. Distribution of dose intensity percent according to CADD-based gene-wise variant burden score groups for *NUDT15*. (regression p-value for trend = 0.011)**


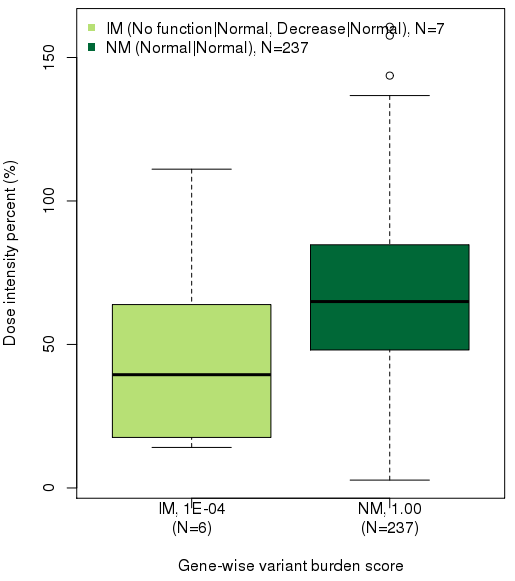


**Supplementary Figure S8. Distribution of dose intensity percent according to CADD-based gene-wise variant burden score groups for *TPMT*. (regression p-value for trend = 0.011)**

## Supplementary Tables

**Supplementary Table S1. Protein-coding SNVs + InDels identified in 244 ALL samples.**

| **GENE** | **POS** | **rsID** | **REF** | **ALT** | **TYPE** | **HGVS.p** | **SIFT** | **CADD** | **Associated star allele** | **Frequency (%),N=244** |
| --- | --- | --- | --- | --- | --- | --- | --- | --- | --- | --- |
| *NUDT15* | 13:48611918 | rs554405994 | AGGAGTC | AGGAGTCGGAGTC | Disruptive inframe insertion | p.Gly17_Val18dup | NA | 14.35 | *2, *6 | 7 (2.87) |
|  | 13:48611918 | rs746071566 | AGGAGTC | A | Disruptive inframe deletion | p.Gly17_Val18del | NA | 10.35 | *9 | 1 (0.41) |
|  | 13:48611934 | rs186364861 | G | A | Missense | p.Val18Ile | 0.02 | 25.6 | *5 | 4 (1.64) |
|  | 13:48619855 | rs116855232 | C | T | Missense | p.Arg139Cys | 0.09 | 22.2 | *2, *3 | 40 (16.39) |
|  | 13:48619856 | rs147390019 | G | A | Missense | p.Arg139His | 0.12 | 24.6 | *4 | 4 (1.64) |
| *TPMT* | 6:18130918 | rs1142345 | T | C | Missense | p.Tyr240Cys | 0.01 | 28.3 | *3A, *3C, *41 | 6 (2.46) |
|  | 6:18134076 | rs75543815 | T | A | Missense | p.Tyr180Phe | 0.78 | 15.28 | *6 | 1 (0.41) |
|  | 6:18139214 | rs2842934 | G | A | Synonymous | p.Ile158Ile | 1.00 | 14.07 | *1S | 223 (91.39) |

Haplotypes were inferred via PHASE2 software. Star allele genotypes were matched using the PharmGKB haplotype translational table.

**Supplementary Table S2. Summary of subjects carrying *NUDT15* and *TPMT* variants**.

|  | *NUDT15* | | | | | *TPMT* | | | Total, N=244 | Dose indensity percent ≤ 25, N=19 | GVB*^NUDT15^* | GVB*^TPMT^* | GVB*^NUDT15,TPMT^* |
| --- | --- | --- | --- | --- | --- | --- | --- | --- | --- | --- | --- | --- | --- |
|  | p.Gly17_Val18dup  (rs554405994) | p.Gly17_Val18del  (rs746071566) | p.Val18Ile  (rs186364861) | p.Arg139Cys  (rs116855232) | p.Arg139His  (rs147390019) | p.Ile158Ile  (rs2842934) | p.Tyr180Phe  (rs75543815) | p.Tyr240Cys  (rs1142345) |  |  |  |  |  |
| SIFT | NA | NA | 0.02 | 0.09 | 0.12 | 1.00 | 0.78 | 0.01 |  |  |  |  |  |
| CADD | 14.35 | 10.35 | 25.6 | 22.2 | 24.6 | 14.07 | 15.28 | 28.3 |  |  |  |  |  |
|  |  |  |  | ✓ |  | ✓ | ✓ |  | 1 (0.41) | 1 (5.26) | 0.30 | 1 | 0.30 |
|  | ✓ |  |  | ✓ |  | ✓ |  |  | 5 (2.05) | 0 (0.00) | 5E-03 | 1 | 5E-03 |
|  | ✓ |  |  | ✓ |  |  |  |  | 1 (0.41) | 0 (0.00) | 5E-03 | 1 | 5E-03 |
|  | ✓ |  |  |  |  | ✓ |  |  | 1 (0.41) | 1 (5.26) | 1E-04 | 1 | 1E-04 |
|  |  | ✓ |  |  |  | ✓ |  |  | 1 (0.41) | 1 (5.26) | 1E-04 | 1 | 1E-04 |
|  |  |  |  | ✓ |  | ✓ |  |  | 30 (12.30) | 4 (21.05) | 0.09 | 1 | 0.09 |
|  |  |  |  |  | ✓ | ✓ |  |  | 4 (1.64) | 0 (0.00) | 0.35 | 1 | 0.35 |
|  |  |  |  |  |  | ✓ |  | ✓ | 6 (2.46) | 2 (10.53) | 1 | 0.1 | 0.1 |
|  |  |  | ✓ |  |  | ✓ |  |  | 4 (1.64) | 0 (0.00) | 0.14 | 1 | 0.14 |
|  |  |  |  | ✓ |  |  |  |  | 3 (1.23) | 1 (5.26) | 0.30 | 1 | 0.30 |
|  |  |  |  |  |  | ✓ |  |  | 171 (70.08) | 8 (42.11) | 1 | 1 | 1 |
|  |  |  |  |  |  |  |  |  | 17 (6.97) | 1 (5.26) | 1 | 1 | 1 |
|  |  |  |  |  |  |  |  | Total | 244 (100) | 19 (100) |  |  |  |
